# The Treatment of Syphilis

**Published:** 1913-08

**Authors:** Edgar G. Ballenger, Omar F. Elder

**Affiliations:** Atlanta, Georgia; Atlanta, Georgia


### THE TREATMENT OF SYPHILIS.

By Edgar G. Ballenger, M. D., and Omar F. Elder.
Atlanta, Georgia.

While we realize that the title of our paper includes a field much to extensive to be covered in the time allotted for the reading of papers, we thought it might be of interest to discuss a few of the salient features of this subject.

Our views regarding the treatment of syphilis have undergone such a revolution during the last few years that unless one has been actively associated with the modern management of this disease he may not fully appreciate the significance of many of the changes. Without discussing the inadequacy of the older treatment with mercury and iodides, or the early treatment with salvarsan, when we hoped that one or two large injections of this remedy would effect a lasting cure, I will outline briefly the course of treatment that at the present is thought necessary in the treatment of syphilis.

First, last and always we must remember that hard and fast rules cannot be laid down. Instead of rules they should be regarded merely as suggestions. In every instance we must consider the individuals characteristics of the patient, his physical condition and vitality, as well as the stage and characteristics of the luetic infection. There is no disease in which the manifestations may be so varied, nor any in which so many parts of human organism may become involved. No organ or part of the body is immune.

We should constantly bear in mind: 1st. The tendency for the disease to remain latent for years only to recur later as typical or atypical syphilis. 2nd. The peculiarities of the disease acquired by the mother in conceptional infection, and of babies born apparently healthy when the mother is diseased. 3rd. The tendency of the spirochetes to cause little disturbance during the primary, secondary and tertiary stages only become active in the fourth stage and produce the so-called parasyphilitis affections. 4th. The insidious attack on the heart and arteries, and many other serious organic changes. 5th.

Such possibilities and probabilities should be kept constantly before us in order to forestall, if possible, serious consequences.

To treat syphilis correctly naturally implies a CORRECT DIAGNOSIS, and this is not always easy to make. Of equal or greater importance in the treatment of chancre is an EARLY DIAGNOSIS, and in after years the RECOGNITION OF LATENT SYPHILIS.

In this part of modern syphilology we have reached a fair degree of accuracy in the application of the various tests as, as search for the spirochaeta pallida with the dark field illumination, the Wassermann reaction of the blood, and spinal fluid, the provocative reaction, the luetin test and the presence of lymphocytes or leucocytes in the spinal fluid. So important is the diagnosis of lues before the secondary outbreak or scattering of the spirochetes throughout the body that in nearly every instance a correct diagnosis should be made by finding the spirochetes or by the positive Wassermann. We should remember the unreliability of a negative Wassermann at all times, and especially before the second or third week. A positive test becomes more and more likely as we approach the sixth week. With our present therapeutic methods whereby we practically stop at once the spirochetal invasion and greatly lessen the future dangers for the patient, the early diagnosis is of much greater importance than it was in presalvarsan days. So, in the treatment of chancre, the early diagnosis becomes of equal importance to the requisite number of injections of salvarsan or neosalvarsan.

During the early secondary period the diagnosis takes secondary place and the prevention of neurocurrences by an intensive course of treatment becomes the chief dissideratum. It is my belief that eye and ear lesions only follow inadequate courses of treatment and are decidedly more likely during this period than the primary or later stages. The danger here comes not from too much salvarsan, if the doses be of medium size and properly spaced, but too little. Our early fear that the spirochetes might become immunized to salvarsan has been entirely dissipated and now we prefer small or medium size doses repeated every one or two weeks until about two and one-half or three grammes of salvarsan or four or five grammes of neosalvarsan have been administered, or more in the occasional cases when the lesions are slow in disappearing or the Wassermann reaction does not become negative. By repeating the medium size doses we both minimize the danger of the remedy and effect a more thorough cure.

There are reasons both for and against the administration of mercury and iodides during this treatment. The danger of neurorecurrences seems lessened by giving the mercury if a full amount of salvaran or accurate and frequent examinations are not possible. On the other hand, the continued administration of mercury may give us a false sense of security and render the Wassermann temporarily negative, thus delaying a more intensive and very necessary course of treatment if our efforts in previous treatment have been inadequate. As a rule, hwver, it is adviseable to give an intensive course eof mercury during the treatment with salvarsan or neo-salvarsan.

If salvarsan or neosalvarsan be preprly given intravenously every one or two weeks and the doses regulated according to the weight and health of the patient, we think the danger is insignificant. Certainly it is trivial when compared with the danger of uncured syphilis fhich is an ever present and imminent danger inseparable from the average conventional treatment with mercury and iodides. These are valuable remedies and there is absolutely no reason why we should pit them against salvarsan, but every reason why all should be scientifically employed to combat the ravages of syphilis. That mercury and iodides alone, as used in the past, are inadequate is amply proven by the frequency of tabetics, paretics, aneurysms, syphilitic cordiac lesions, and many other late eye, ear, nose and throat lesions. While referring to the last mentioned affections I would like to call the attention of the eye, ear, nose and throat specialists to the responsibility placed upon them in dealing with these manifestations of uncured syphilis. Although many realize the character and significance of these lesions, some are quite content to heal the lesion without giving the full and adequate course of treatment that is so urgently demanded to exterminate the latent disease in order to prevent parasyphilis.

These affections are red flags warning of imminent danger but the patient of course does not realize the significance of these tertiary manifestations, and the physician who originally treated the early syphilis may not have seen the patient for years. These lesions may be the only warnings bebetween the primary and secondary periods and the fourth stage or parasyphilis which has been shown to be modified syphilis, and caused by the spirochaeta pallida. The onset may be insidious and serious damage beyond repair may occur before the patient realizes his need of treatment.

To merely heal the lesions and not treat the patient until the Wassermann reaction remains permanently negative is like throwing away the red flag that warns the engineer of the danger ahead. It is in this or similar stages of syphilis that we should tieat the brain and nervous lesions or at their incipiency. In advanced paresis or locomotor ataxia more than the usual care must be exercised in regulating the doses.

All patients should be urged to drink freely of water the day salvarsan or neosalvarsan is administered, and four or five days later so as to dilute the remedy as it is eliminated and thus prevent irritation of the kidneys.

No patient should be declared well until his blood and spinal fluid remain permanently negative to the Wassermann reaction and the spinal fluid shows by its cell content that disease is absent.

---

If a radiograph of the unemptied bladder, exposed with the patient in the level supine or reversed Trendelenburg position, shows a shadow or group of shadows in the region of the neck of the bladder, and a second radiograph, exposed with the patient in the Trendelenburg position and the x-rays passing in the same relative direction, shows the shadow in the same place as before, the stonne or stones are fixed in the prostate or the prostatic urethra or in a diverticulum behind the prostate.—American Journal of Surgery.

